# Supplementary material for: Optimizing learning outcomes in physical education: A comprehensive systematic review of hybrid pedagogical models integrated with the Sport Education Model
Source: PLoS One. 2024 Dec 4;19(12):e0311957. doi: 10.1371/journal.pone.0311957 (PMC11616820; doi:10.1371/journal.pone.0311957)
Supplement: S1 Table — (DOC) [file pone.0311957.s001.doc]

| **S1 Table. Detailed search strategy** | |
| --- | --- |
| Search database | keyword |
|  | TITLE-ABS-KEY ("Sport Education") AND ("pedagogical model" OR "curriculum model" OR "instructional model" OR "physical education" OR "hybrid*" OR "Integrate*" OR "combine*") |
| Web of Science | n = 557 |
| Scopus | n = 696 |
| PubMed | n = 36 |
| EBSCO host | n = 109 |
